# Energetics for CO_2_ Reduction by Molybdenum-Containing Formate Dehydrogenase

**DOI:** 10.1021/acs.jpcb.2c00151

**Published:** 2022-02-22

**Authors:** Per E. M. Siegbahn

**Affiliations:** Department of Organic Chemistry, Arrhenius Laboratory, Stockholm University, SE-106 91 Stockholm, Sweden

## Abstract

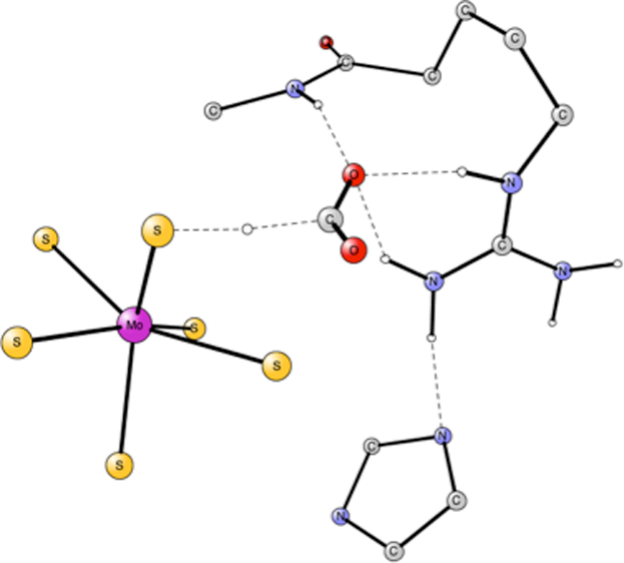

The level of carbon dioxide in the atmosphere has increased in a dangerous way during the past century. Methods to decrease this level are therefore of high interest at present. Inspiration to do so in an efficient way could come from biological systems. Molybdenum-containing formate dehydrogenase (Mo-FDH) is one of the most interesting enzymes in this respect. For example, the reduction potential required is not very low. The normal reaction catalyzed by Mo-FDH is actually the opposite one of oxidizing formate to CO_2_. However, recent electrochemical studies have shown that the reaction can be reversed by a moderate lowering of the reduction potential. The goal of the present study has been to study the full mechanism of Mo-FDH, particularly in the most interesting direction of reducing CO_2_, which has not been done before. The methods used are the same as those that have been shown to give excellent results for redox enzymes in all cases they have been tested. The results obtained for Mo-FDH are also in excellent agreement with the experimental results.

## 1 Introduction

**Figure 1:**
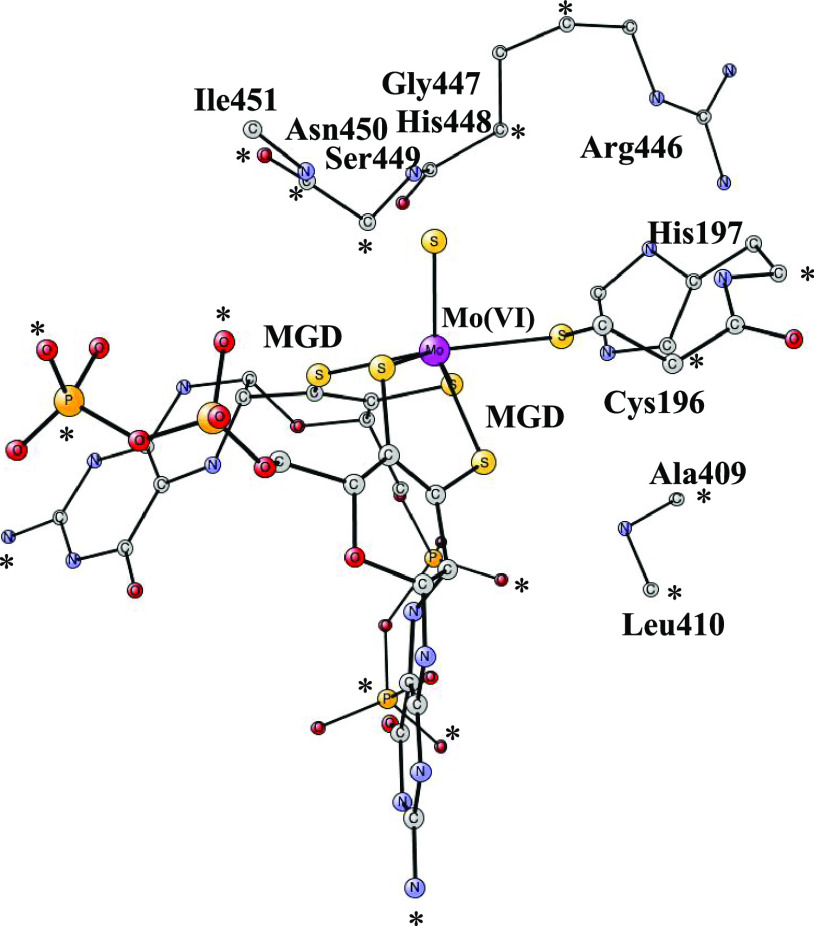
Model used for the active site of Mo-containing formate dehydrogenase Mo-FDH, illustrating which atoms were included. Starting coordinates were taken from PDB entry 1KQF.^6^ H-atoms are removed. Atomic positions frozen from the X-ray structure are indicated by an asterisk.

Carbon dioxide fixation in nature is mainly performed by the enzyme Rubisco.^1^ The process is very slow and unspecific. Because of the increasing levels of carbon dioxide in the atmosphere, there is at present a large interest to find alternatives. One of the most interesting alternatives is to use electrons and protons produced by sunlight and water in photosynthesis, to reduce CO_2_. A key to the success of such an approach is to avoid the formation of hydride ions that will eventually lead to H_2_ formation rather than the reduction of CO_2_. Reversible formation of CO from CO_2_ is in nature accomplished by CO dehydrogenases.^2^ There are two types of this enzyme. In one of them, Ni-CODH, a tetranuclear NiFe_3_ catalyst is used. The mechanism for this enzyme has recently been studied by density functional theory (DFT) using a cluster modeling of the active site.^3^ The experimental redox potential for oxidation is −0.32 V.^4,5^ The second one is Mo, Cu-CODH. In the present study, the same techniques are used for another enzyme that activates CO_2_, namely, molybdenum-containing formate dehydrogenase (Mo-FDH) that uses a mononuclear molybdenum complex as the catalyst, see Figure 1.^6^ The overall reaction of Mo-FDH can be written as follows:
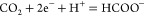


The forward reaction in the enzyme takes formate and produces CO_2_, protons, and electrons. The mechanism of this reaction has been studied theoretically before,^7−14^ but of main interest in the present study is instead the reverse reaction forming formate from CO_2_. The reversibility of the reaction has been demonstrated using electrochemical methods. Perhaps surprisingly, the reaction became reversible with a quite high redox potential of −0.4 V.^4^ The present investigation has been inspired by that study. In comparison to the present system, the reduction of N_2_ by nitrogenase requires a redox potential of −1.6 V. When the reversibility of Mo-FDH is studied, it is necessary to consider also the steps where electrons and protons are added/removed. These steps have been avoided in previous studies, but they are addressed here.

Five different mechanisms have been suggested for the forward reaction of Mo-FDH in previous studies.^7−14^ In the most recent study by Dong and Ryde,^14^ they have all been tested, and one of them was suggested as the most plausible. In this mechanism, formate enters the active site and does not coordinate to Mo. The present study of the forward reaction is in full agreement with that suggestion. In all the other suggestions, formate was coordinated to Mo. In some of them, a sulfur shift forming an S-S bond was suggested.^8−10^

## 2 Methods

The methods used here are the same as the ones used recently for many other redox enzyme mechanisms; see below.^15^ Very similar methods have also been used previously for other enzyme mechanisms.^16,17^ The starting point is the standard B3LYP method,^18^ which has 20% exact exchange. It has been found that the B3LYP results are almost only sensitive to the exact exchange part.^15−17^ The key for obtaining an estimate of the accuracy obtained is therefore to vary this fraction from 10% to 15% to 20%. So far, the best agreement with experiments for enzyme mechanisms has been obtained for a fraction of about 15%.

The redox enzyme mechanisms where the methodology was tested^15^ were the following: photosystem II, nitrogenase, cytochrome c oxidase, NiFe and FeFe hydrogenases, NiFe-CO dehydrogenase, multi-copper oxidases, and acetyl-CoA synthase. In multi-copper oxidase and cytochrome c oxidase, O_2_ is cleaved. Photosystem II forms O_2_, nitrogenase cleaves N_2_, and they have cofactors with many transition metals. FeFe hydrogenase, NiFe-CO dehydrogenase, and acetyl-CoA contain FeS clusters. However, because DFT is a single determinant method, it means that it will fail in strong multireference cases. The question is only where the limit is between single-reference and multireference cases. Because the above tests for the redox mechanisms show that the methodology works very well for all these redox enzymes, which are the only ones tested so far, the conclusion is that the ground states of these enzymes are not strong multireference cases. The failures reported for some of these systems are probably due to the fact that highly excited structures were used, in the case of NiFe hydrogenase 40 kcal/mol above the lowest state.^19,20^

The geometries were optimized using an LACVP* basis set, which is of a moderate DZP size. For the final energies, a larger basis set was used with cc-pvtz(−f) for the nonmetal atoms, and with LAV3P* for the metals. These calculations had to be done without the pseudo-spectral approach. Solvation effects were obtained using a Poisson–Boltzmann solver,^21^ with a dielectric constant of 4.0. Zero-point effects were obtained from computed Hessians with the LACVP* basis. D3 dispersion was included in the geometry optimization, and D2 was used for the final energies.^22^ Translational entropy effects of 9.6 kcal/mol (obtained from a particle in a box) were included in the steps where CO_2_ was bound or released. In the other steps, entropy effects were assumed to be small. The calculations have been performed with the programs Jaguar^21^ and Gaussian 09.^23^

The present model with altogether 160 atoms was constructed based on the PDB entry 1KQF;^6^ see Figure 1. The model includes all residues that could possibly affect the mechanism by, for example, hydrogen bonding. There are two bidentate molybdopterin guanine dinucleotide (MGD) ligands, one selenocysteine (Sec196) ligand and one sulfido ligand bound to molybdenum, forming an octahedral complex. The model for the two MGD ligands includes their two phosphate groups but is truncated after the second one. The tetrahydro form was used for the pterin. The outermost phosphates are doubly protonated, and two oxygens are frozen from their X-ray positions. The innermost ones are singly protonated and one oxygen position is frozen. Selenium is replaced by a sulfur. In the second sphere, the model contains Arg446, His197, the backbone from Arg446 to Ile451 (Gly447, His448, Ser449, and Asn450), and the backbone between Ala409 and Leu410. The frozen atoms are indicated by an asterisk in Figure 1**.** The principles used for freezing atoms are that they are either backbone atoms or atoms strongly hydrogen-bonded to atoms outside the model. The full details of the model including the atomic positions that were frozen are given in the Supporting Information.

The present study of the mechanism includes also, for the first time, the details of the reduction steps. These steps are here shown to have a significant effect on the rate-limiting step. For the forward oxidation reaction, the experimental redox potential of −0.32 V was used, while for the reverse reduction reaction, the experimental limiting redox potential of −0.4 V was used.^4^ This means that for the values used in the calculations using the standard hydrogen electrode potential (4.281 eV), 91.4 kcal/mol for the forward oxidation and 89.5 kcal/mol for the reverse reduction reaction were applied. For the protonation steps, the experimental value for a proton in water was used, 279.8 kcal/mol for pH = 7.^24^

## 3 Results

The mechanism for the oxidation of formate has been studied previously using quantum chemical methods,^7−14^ but not the one for the reduction of CO_2_. The individual oxidation steps were not considered either in the previous studies.

An important difference to Ni-CODH, which can also activate CO_2_ and which has been studied before using similar methods,^3^ is that the product for Mo-FDH is a formate anion, while in Ni-CODH, it is a neutral CO. Therefore, the overall reaction for Mo-FDH involves a different number of electrons and protons. That makes this enzyme different from almost all the other ones discussed in our previous studies. When a (H^+^,e^–^)-couple is added to the cofactor, there is no change of charge and therefore no long-range electrostatic contribution to the energy. When just an electron is added, the electrostatic effects may extend past the borders of the cluster model used because the charge changes. An estimate of these long-range effects from the outside of the model to the computed redox potential is therefore needed, in general. The best way to obtain this estimate is to use an experimentally determined value related to a proton-uncoupled redox potential. This was the procedure successfully used for PSII.^25^ In this case, the estimate of the long-range electrostatic contribution was 5.6 kcal/mol. In the present case, the experimental value for the limiting redox potential is −0.4 V, for which the reaction becomes reversible^4^ can be used to obtain the long-range contribution. Rather surprisingly, the long-range correction becomes close to zero. With a redox potential of the reductant of −0.4 V, derived from the electrochemical experiments, the reduction becomes endergonic by +0.2 kcal/mol. For the oxidation reaction, the experimental value of −0.32 V led to an exergonicity of −4.0 kcal/mol. By a small change of 0.1–0.2 V of the redox potential, the reaction becomes reversed.

The main focus of the present study is on the reverse reaction producing formate from CO_2_, which is of the most current interest because of its possible use in reducing the CO_2_ level in the atmosphere. In the present section, the results for this reaction are discussed first. The forward oxidation of formate to CO_2_ is described afterward.

### 3.1 CO_2_ Reduction

The starting point for the reduction of CO_2_ is the structure shown in Figure 1**.** A notable feature is that Arg446 and His197 are bound together by a very strong H-bond with a distance of only 1.78 Å. The positive Arg446 is a key residue for the mechanism; see below. CO_2_ is not yet bound, and the charge of the model is zero. The calculation of the electronic structure converged to a closed-shell Mo(VI) singlet state, starting from an open-shell singlet. Surprisingly, the triplet state is only slightly higher in energy by 1.0 kcal/mol, after all corrections are added. This is the only structure for which the singlet is not markedly lower than the triplet. The first step of the mechanism is an addition of a (H^+^,e^–^) couple. This does not lead to any long-range electrostatic effects because the addition is neutral. The lowest energy is obtained if the proton is added to the sulfido ligand. The spin is localized on Mo, indicating a Mo(V) state. For a redox potential of −0.4 V, the reduction is exergonic by −10.0 kcal/mol. The product state becomes the resting state for the reduction; see further below. The charge of the model is still zero.

In the second step, the substrate CO_2_ enters. The best position is binding to the sulfide of Cys196. With the loss of translational entropy of 9.6 kcal/mol, the binding becomes endergonic by +6.1 kcal/mol. There are two strong stabilizing H-bonds with one from Arg446 to one of the oxygens of CO_2_ with a distance of 1.77 Å, and with one to the other oxygen from an N–H backbone of 1.87 Å. There is also a weaker one from another N–H backbone group, with a distance of 2.18 Å. A search for a transition state indicated that there may be an additional small barrier for the binding of 2–3 kcal/mol. There is a change in the wavefunction as CO_2_ binds. Before binding, the spin is on Mo, leading to a Mo(V) complex, as mentioned above. After the binding, the spin becomes very delocalized over the ligands on the Mo complex. The spins on the different atoms of the ligands are generally smaller than 0.10 with an exception for a nitrogen, which has a spin of 0.25. The spin on Mo is zero, indicating a Mo(IV) state. There is an enthalpic binding with a C–S distance of 1.90 Å. However, the binding is not strong enough to compensate for the loss of entropy of 9.6 kcal/mol.

**Figure 2:**
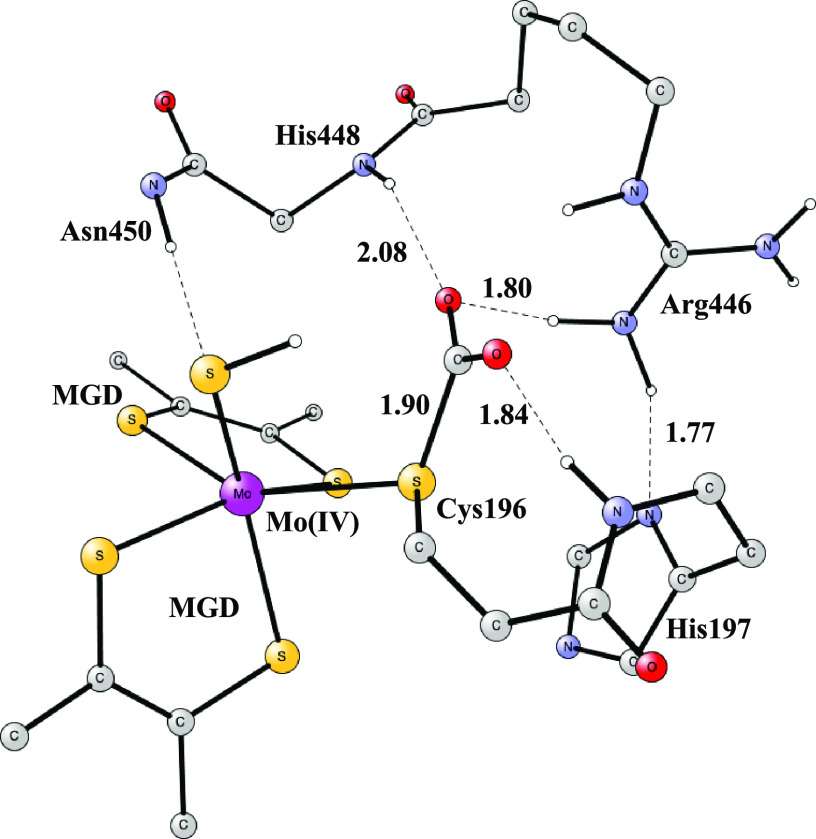
Binding of CO_2_ to Cys196 after the addition of a proton and two electrons.

In the third step, an electron is added to the complex. With the redox potential of the reductant of −0.4 V, the addition becomes exergonic by −3.1 kcal/mol. The structure in Figure 2 is obtained, which is quite similar to the one obtained in the previous study by Dong and Ryde.^14^ The wavefunction is now that of a closed-shell Mo(IV) complex. The triplet state is 9.0 kcal/mol higher in energy. Even though there is a large change in the spin distribution on the Mo complex after the addition of the electron, the structure hardly changes, which is favorable for a fast electron transfer. The same H-bonds are present as the ones before the electron transfer. The bond between Arg446 and one of the oxygens of CO_2_ is now 1.80 Å, the one from the backbone to the other oxygen is 1.84 Å, and the weaker one from another backbone is 2.08 Å.

**Figure 3:**
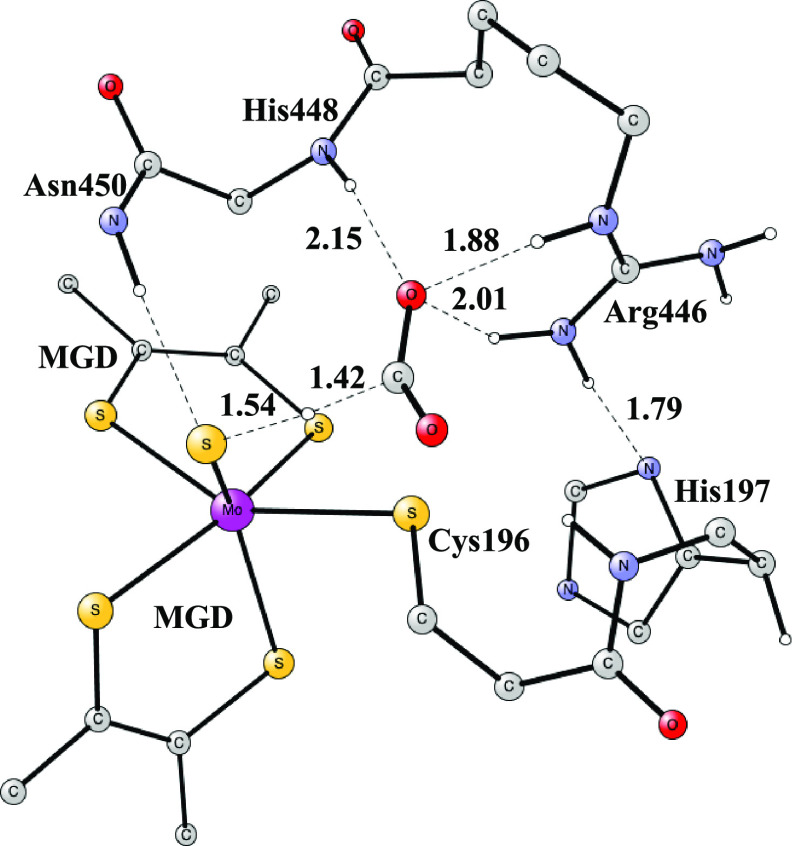
Transition state for hydride transfer to CO_2_.

In the fourth step, there is a hydride transfer to CO_2_ with a proton from the sulfide ligand and two electrons from molybdenum, yielding a Mo(VI) complex. The optimized transition state is shown in Figure 3. The hydride distance to the sulfide is 1.54 Å, and the one to CO_2_ is 1.42 Å. There is no spin anywhere on the structure. The H-bonds to CO_2_ are quite similar to the ones in the reactant; see above. The most notable change is that the orientation of Arg446 changes so that one H-bond to CO_2_ increases from 1.84 to 2.01 Å and another one shortens from 2.20 to 1.88 Å. The barrier is 16.0 kcal/mol.

**Figure 4:**
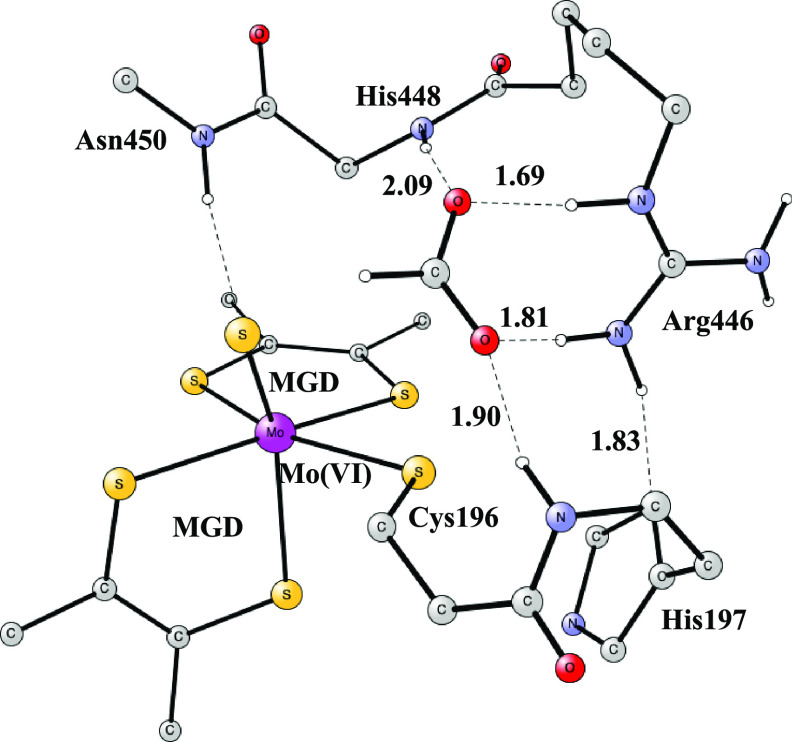
Binding of the formate product. This structure has an energy of +0.2 kcal/mol, as shown in the scheme in Figure 5.

After the TS, the formate product is bound, as shown in Figure 4. The closed-shell singlet state is lowest with the triplet 10.0 kcal/mol higher in energy. There are two strong H-bonds to Arg446 with distances of 1.69 and 1.81 Å. There is also a weaker one to the backbone of His448 of 2.09 Å. From the bound S–CO_2_ reactant shown in Figure 2, the energy goes up by +7.2 kcal/mol.

For the CO_2_ reduction, it is very important that no Mo-hydrides are formed during the catalytic cycling. With Mo-hydride formation, it is very difficult to avoid the competing reaction of forming H_2_. This makes the present mechanism particularly interesting from a technical perspective.

**Figure 5:**
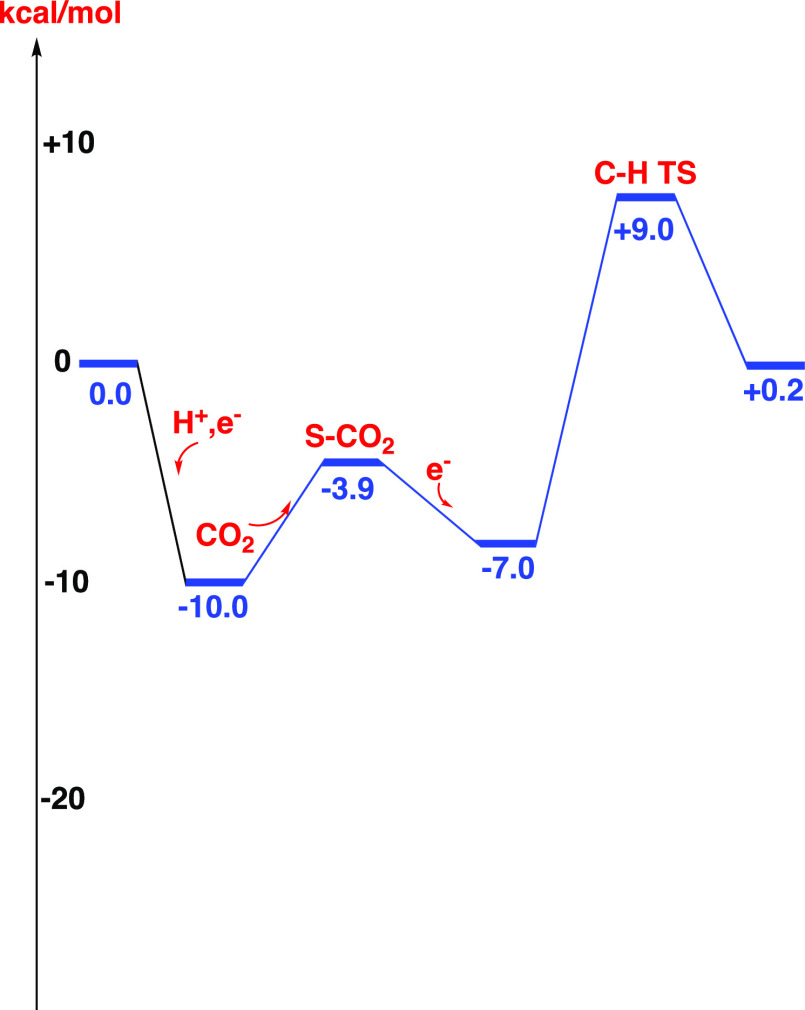
Energy diagram for CO_2_ reduction to formate.

The energy diagram for CO_2_ reduction is shown in Figure 5. The resting state is the one where an (H^+^,e^–^) has been added to the complex before CO_2_ has become bound. From this point to the hydride-transfer TS, the barrier is 19.0 kcal/mol, which is rate-limiting. The overall reaction energy is +0.2 kcal/mol, which is in excellent agreement with the electrochemical experiment of 0.0 kcal/mol.^4^ The local barrier from the bound CO_2_ reactant is 16.0 kcal/mol, showing that the reduction steps increase the barrier by +3.0 kcal/mol. By reducing the redox potential of the reductant to −0.5 V, the reduction becomes exergonic by −4.4 kcal/mol, and the rate-limiting barrier decreases to +16.7 kcal/mol.

### 3.2 Formate Oxidation

The forward reaction for Mo-FDH is the oxidation of formate to CO_2_. Most recently, this reaction has been studied by Dong and Ryde.^14^ The present study leads to the same mechanism as was suggested in their study, but a difference is that also the oxidation steps are included here. The complete reaction cycle has not been studied before. It is of interest to investigate to what extent the oxidation steps modify the rate-limiting barrier.

The first step in the oxidation reaction is to bind formate. In the previous study by Dong and Ryde, it has been shown that formate does not bind as a ligand to molybdenum.^14^ This is very important for the possibility of making the reverse reduction reaction possible; see above. If there would have been a possibility to form a direct bond to molybdenum, it would have been difficult to avoid hydride, and thereby H_2_, formation rather than forming formate.

The optimal binding site for formate is shown in Figure 4. There has been a suggestion by Cerqueira et al. termed the “sulfur-shift mechanism,” in which an S–S bond is formed between the sulfido and the Cys196 ligands.^9^ The formation of an S–S bond has been tested here with the present larger model, and it indeed turned out to be a possibility. The S–S bond was formed with a small barrier and with essentially the same energy as for the formate reactant shown in Figure 4. The product has a triangular bidentate S–Mo–S structure, with short bond distances between Mo and the sulfurs of 2.39 and 2.68 Å, respectively. The S–S bond distance is 2.11 Å. However, in the sulfur-shift mechanism, S–S bond formation was suggested to be followed by a release of the S–S complex from molybdenum, creating an open site where the formate substrate could bind to Mo. In the previous study by Dong and Ryde, a very high energy for the structure with formate bound to molybdenum was obtained,^14^ and the mechanism was rejected. A very similar result was obtained here. The reason for the high energy is that there will be very short distances to the peptide atoms surrounding the active site. These atoms were missing in the small model used by Cerqueira et al.^7^ The question of creating an open site on molybdenum is also of interest in the context of forming hydrides. Because the creation of open sites on molybdenum has a high energy cost, hydrides bound to molybdenum cannot easily be formed, and unwanted H_2_ formation is, therefore, to a large extent, avoided.

After the outer sphere binding of the formate, the reaction goes directly to the TS, as shown in Figure 3. In this process, the oxidation state changes from Mo(VI) to Mo(IV). The barrier is quite low with 8.8 kcal/mol. In the study by Dong and Ryde, a very similar barrier of 6.7 kcal/mol was found in their QM/MM study using B3LYP. Interestingly, with a big QM model of 1161 atoms using the TPSS functional, the barrier became as low as 3.8 kcal/mol. From their results, it seems that the main reason for the discrepancy is that the functionals used in the big QM and in the QM/MM calculations were different.

Like in the study by Dong and Ryde, the CO_2_ product after the TS does not directly leave the complex but binds to the sulfur of Cys196. In order to release CO_2_, an electron needs to leave the complex. Oxidation leads to a small endergonicity of +1.2 kcal/mol. Afterward, CO_2_ can leave with a low barrier in an exergonic step by −6.1 kcal/mol. To complete the catalytic cycle, an (H+,e−) needs to leave the complex, which is endergonic by +8.1 kcal/mol. The energetics of these steps has not been studied before.

**Figure 6:**
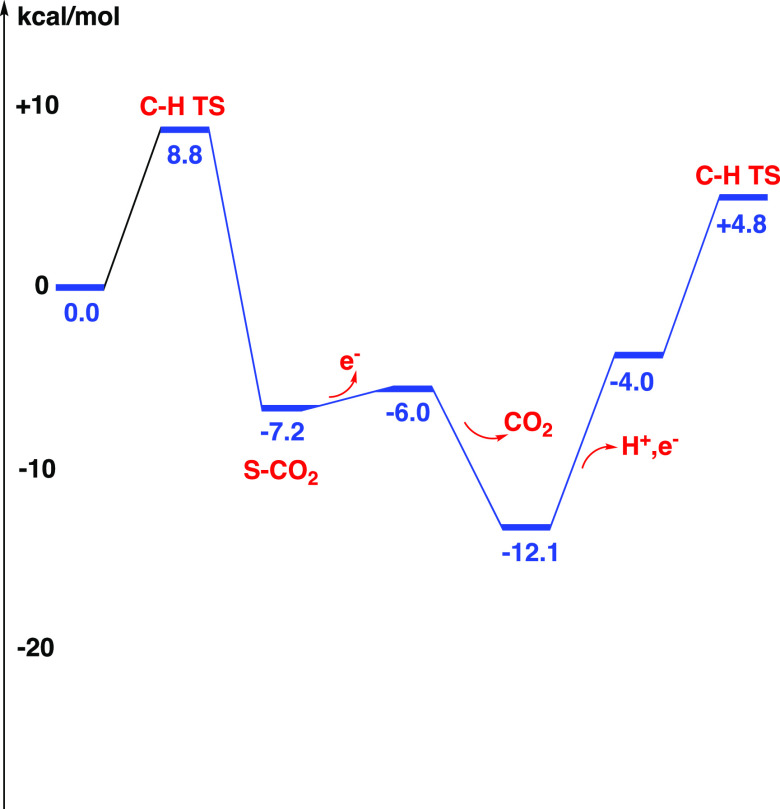
Energy diagram for the oxidation of formate to CO_2_.

The energy diagram for the oxidation reaction is shown in Figure 6. The beginning of the next cycle is also shown in the figure. The most interesting part of the diagram is the rate-limiting barrier of the process. The resting state is the point where CO_2_ has left at −12.1 kcal/mol. After that, the release of (H+,e−) occurs, which is endergonic by +8.1 kcal/mol. In the next step, there is the hydride-transfer TS with a local barrier of +8.8 kcal/mol. This means that the rate-limiting barrier becomes (8.1 + 8.8) = +16.9 kcal/mol, which is much higher than the local barrier of only +8.8 kcal/mol for hydride transfer. This shows the importance of considering also the oxidation steps of the cycle, not just the chemical steps.

## 4 Discussion and Conclusions

Both the reduction of CO_2_ to formate and the reverse oxidation from formate to CO_2_ have been studied using methods proven to be very accurate for many other redox enzymes.^15−17^ The energies for the redox steps have been calculated, which leads to the possibility of studying the reversibility of the reaction, which had not been done before. By using the standard value for the binding of a proton in water and the experimental limiting redox potential, an excellent agreement was obtained for the limiting value of the redox potential for reversing the reduction reaction, which has been measured to be −0.4 V.^4^

The reduction of CO_2_ has been the main focus of interest in the present study because of its possible use of fixing CO_2_ in the atmosphere. The resting state of the reaction is obtained after an exergonic addition of an (H+,e−) to the sulfide ligand of the Mo(VI)-complex. The resulting Mo(V) complex is able to bind CO_2_, but the binding is endergonic. A bond is formed between carbon in CO_2_ and the sulfur of Cys196. The binding is stabilized by an additional reduction, which is not proton-coupled. The modeling of such a process can be difficult, but in the present case, it turned out to be straightforward. The reason is that the redox potential of the complex does not have significant long-range contributions from the region outside the model. In the next step, there is a hydride transfer, which is part of the rate-limiting step. The local barrier is +16.0 kcal/mol. After the TS, the formate product is released. However, because the reactant is +3.0 kcal/mol higher than the resting state, the rate-limiting barrier becomes +19.0 kcal/mol. By lowering the reduction potential to −0.5 V, the barrier is decreased to +16.7 kcal/mol, and the reaction becomes exergonic by −4.4 kcal/mol. It is shown that the difficulty to create an open site on molybdenum leads to the avoidance of hydride formation, thereby also avoiding the competing reduction of H_2_ formation.

The effects of the redox steps become even more pronounced for the reverse reaction of formate oxidation. The local barrier for the release of the hydride is +8.8 kcal/mol, but the rate-limiting barrier becomes +16.9 kcal/mol. The reason is that the resting state is not the one just before the hydride transfer, but is instead at the state where CO_2_ binds to the sulfur of Cys196.

## Supporting Information Available

Coordinates for all structures appearing in the mechanism (PDF)
