# Supplementary material for: Energetics for CO2 Reduction by Molybdenum-Containing Formate Dehydrogenase
Source: J Phys Chem B. 2022 Feb 22;126(8):1728–33. doi: 10.1021/acs.jpcb.2c00151 (PMC8900120; doi:10.1021/acs.jpcb.2c00151)
Supplement: Supplementary file 1 — jp2c00151_si_001.pdf [file jp2c00151_si_001.pdf]

Supporting information:

The Energetics for CO_2 reduction by Molybdenum-Containing Formate Dehydrogenase

Per E. M. Siegbahn*

Department of Organic Chemistry, Arrhenius Laboratory, Stockholm University, SE-106 91, Stockholm, Sweden

*Corresponding author: per.siegbahn@su.se

Coordinates for the structure in **Figure 2.** Frozen positions marked with #.

Mo1	3.7195295470	47.2956998182	113.8968849261
S2	4.4807706563	47.9596446617	116.1679933985
C3	4.4281363405#	42.6296475254#	114.9024042483#
C4	4.5325095561	44.0630311113	115.4706538651
S5	5.3718202524	45.2477975865	114.3106367616
N6	6.7786850877	43.8480748340	107.9927190259
C7	5.6899656049#	42.8799862208#	107.7290038951#
C8	10.6099994665#	48.2449997719#	113.6600004305#
C9	11.4888296591	48.5735890026	114.8631832132
C10	11.6430398341#	47.4500620313#	115.8922558340#
C11	11.7893508842	46.0195938645	115.3361421107
N12	10.4997575472	45.4910679835	114.8584829290
C13	10.3060577361	44.8788587355	113.6807232042
N14	11.2885735339	44.1050218412	113.1560016024
N15	9.1739069087	45.0273824581	113.0080574893
P16	-2.7370399193#	52.6929821654#	116.1180039211#
O17	-3.3869546592	53.4479164886	115.0386167344
O18	-1.1433046138	52.6905147333	116.1706975992
O19	-3.1101198485	51.0881986667	116.0211384259
O20	-2.6023685838	48.6299325295	115.7789856920
P21	-2.8274352616	49.7468469693	116.8855274819
O22	-1.3500388633#	50.1039769186#	117.5020069045#
O23	-3.8295977565	49.3953625013	117.9063146514
O24	-3.1090411150#	53.1509818708#	117.6020050111#
C25	-1.4813248882	48.6182071674	114.8350538446
C26	-1.1128498689	47.1587660976	114.5539373345
O27	-1.9788456400	46.6814451962	113.5229139470
C28	0.3548248114	46.9145935415	114.1913418234
S29	1.6141522324	47.8739021994	114.9276751540
C30	0.6932110894	45.8903346055	113.3685203466
S31	2.3777713821	45.5405270101	113.0022975177
C32	-0.3354038321	45.1074617151	112.6006658799
N33	-0.2791992483	45.5163144893	111.2020372799
C34	-1.4052464134	45.2472185649	110.4187679189

C35	-1.2962682242	45.3559515317	108.9887727241
O36	-0.3396894909	45.7553148641	108.3333146083
N37	-2.4947815679	44.9666906763	108.3242839668
C38	-3.6066259243	44.5118796691	108.9689317061
N39	-4.6870357554#	44.1459811033#	108.1700061953#
N40	-3.7005174043	44.4409947237	110.2682032307
C41	-2.6033702691	44.8598971184	110.9836891871
N42	-2.7317557164	44.7725128988	112.3625690931
C43	-1.7340227345	45.3001418651	113.2375689229
P44	3.6439641480#	47.3369869876#	104.8320080589#
O45	2.7116881486	48.3977192649	104.4225315367
O46	3.2506852232	46.4215889171	106.0825455122
O47	5.1108438053	47.9407300179	105.2731750676
O48	6.3558566161	48.3024595792	107.5132057120
P49	6.4328986844	47.4993564344	106.1338250464
O50	6.0409648272#	45.9839856320#	106.4790041824#
O51	7.6933179551	47.7376879589	105.4099467812
O52	3.9469640163#	46.2479860449#	103.7250059603#
C53	5.3749094232	48.0930309238	108.5610995740
C54	5.7655949335	49.0165868071	109.7180302760
O55	5.5890468109	50.3294906301	109.2083543867
C56	4.9884970779	48.7814737688	111.0142189988
S57	4.9536619281	47.1904318306	111.7547950190
C58	4.3983050623	49.8079768005	111.6752384095
S59	3.6199485648	49.5843595542	113.2271550077
C60	4.4071058595	51.2138896124	111.1262263833
N61	3.1546377225	51.5046487032	110.4278701212
C62	3.2034849739	52.3997035470	109.3558141670
C63	1.9882869844	52.6823878243	108.6419452091
O64	0.8887312753	52.1550069097	108.8018730057
N65	2.1650755623	53.6610300461	107.6256093301
C66	3.3601643510	54.2589424011	107.3459185678
N67	3.3729610824#	55.1939841366#	106.3070078698#
N68	4.4629637228	53.9602964026	107.9708831085
C69	4.3803558682	52.9864898252	108.9350624495
N70	5.5814348451	52.6365773620	109.5435697789
C71	5.6294643462	51.3652123660	110.1954638941
H72	2.6091633435	55.8623384489	106.3391038477
H73	4.2718642950	55.6628569740	106.2748649606
H74	1.3537928959	53.8092102933	107.0370061069
H75	2.5550546119	50.7040090523	110.2620360485
H76	6.3966363374	52.8796963782	108.9931070233
H77	6.5607462493	51.2999536230	110.7737839476
H78	4.5378987930	51.9289981188	111.9491526705
H79	-4.0548880106	53.3614113953	117.6765386933
H80	-0.7841765314	51.9461470172	116.6934902560
H81	-1.2136344470	49.6513479781	118.3503878458
H82	-1.8014562478	49.1155617042	113.9169762170
H83	-0.6345326936	49.1467097642	115.2739807388
H84	-1.3240008592	46.6032196936	115.4859565293

H85	-3.6839182450	44.7385736276	112.7018305130
H86	0.1513291716	46.4205390399	111.0404024886
H87	-4.4438509655	43.6194292521	107.3379366043
H88	-5.3998765921	43.6799737701	108.7197678250
H89	-2.4871580297	45.1109503735	107.3211332747
H90	-0.1134160895	44.0304526132	112.6537883821
H91	-1.7747256254	44.7456511866	114.1908754150
H92	4.0483536211	45.9591140782	106.4184603879
H93	3.9590301418	46.6489871154	102.8406000528
H94	6.5537682520	45.3065912292	107.0384569963
H95	4.3766451753	48.3536008293	108.1960712043
H96	5.3917164698	47.0449396283	108.8760493022
H97	6.8370746754	48.8452042901	109.9287304375
H98	5.0446477723	52.5703110185	115.8809916984
H99	5.3465307906	51.5251577132	117.2962568254
H100	5.6107357025	49.9265257286	115.6236627394
H101	3.5328950703	44.4736764404	115.6142807955
H102	5.0646994939	44.1227777590	116.4232383310
H103	8.5022139563	45.6993793632	113.3998839705
H104	8.7304690721	44.2508593532	112.4682839971
H105	11.2521731452	43.8463098808	112.1821957905
H106	12.0855556124	43.8260856468	113.7048342764
H107	9.6479861642	45.9011531921	115.2495107252
H108	12.4993844982	46.0062465516	114.5003509201
H109	12.1833686941	45.3606179008	116.1204122673
H110	12.5129897664	47.6487099907	116.5311472098
H111	10.7755437873	47.4473016702	116.5678926904
H112	12.4778405586	48.8670858300	114.4892840132
H113	11.0904628544	49.4516196400	115.3842762250
H114	9.6845949575#	47.6361010334#	113.7923128565#
H115	11.1730293382	47.8760574308	112.8002865304
H116	5.7184857405	42.0220615619	108.4167277876
H117	4.7263264060	43.3879233455	107.8303751058
H118	6.6496114497	44.1974843583	108.9417987074
C119	8.1079993256#	43.2150001196#	107.9359968537#
C120	6.8760003972#	51.0000002181#	114.3990022159#
N121	6.1419513196	50.7934593153	115.5227195601
C122	5.7769597848#	51.9129785355#	116.3690104204#
H123	6.6646778716	52.5108287559	116.5941032617
H124	8.8715190761	43.9593981957	108.1801435843
H125	8.2899912506	42.8583045954	106.9173670742
H126	8.1989900518	42.3668642064	108.6318855456
H127	5.7796046617	42.5173675035	106.7005786824
H128	4.4127793609#	42.6968453811#	113.9047849722#
H129	3.4817622182	42.1777457808	115.2200700152
C130	5.5316279926	41.6397958434	115.1067551838
O131	5.3770115118	40.4437318409	114.8495668802
N132	6.7338231663	42.2103512207	115.3343784850
C133	7.8949985795#	41.6539995791#	114.7259982769#
C134	7.7273649643	41.1143139457	113.2651930608

C135	6.9270003079	42.0077668541	112.3639024122
N136	7.3971645816	43.2291109158	111.9091194978
C137	5.6087967258	41.8746882416	111.9946084508
C138	6.3715562077	43.8416646202	111.3395134073
N139	5.2703697495	43.0458398054	111.3415091707
H140	6.8007940016	43.1869202037	115.6145303554
H141	8.6502163056	42.4486266814	114.7238109835
H142	8.3126019985	40.8265451808	115.3186929913
H143	8.7381586491	40.9669680268	112.8618520782
H144	7.2386222533	40.1399965821	113.3120369183
H145	4.3326199348	43.3560635097	111.1135249145
H146	6.3443494545	44.8708829300	111.0058016710
H147	4.8974234971	41.0898245104	112.1974635401
N148	8.4705315880	49.2610916596	113.9038041282
C149	7.1469997324#	49.7699998156#	113.5680007145#
O150	7.3503444602	52.0967119962	114.1007866895
C151	9.6377664438	49.3967944385	113.1828834255
O152	9.8160526499	50.1758662704	112.2619357628
H153	8.4125339072	48.5013907694	114.5673776974
H154	6.4305045146	48.9638790860	113.7278416619
H155	7.1410284671	50.0556365836	112.5159001310
H156	5.5924964624	47.2227901084	116.3882421892
C157	6.9860914880	45.6389655872	115.2306390772
O158	7.6303132142	46.5797612159	114.7052950655
O159	7.2960355371	44.8787125919	116.1542160016

Coordinates for the structure in **Figure 3.**

Mo1	3.7992020000	47.2121900000	113.9119060000
S2	4.5941980000	47.8677650000	115.9783110000
C3	4.4281360000	42.6296470000	114.9024040000
C4	4.4865080000	44.0892570000	115.4249850000
S5	5.3513130000	45.2088230000	114.2122680000
N6	6.7808060000	43.8543240000	107.9575680000
C7	5.6899660000	42.8799860000	107.7290040000
C8	10.6099990000	48.2450000000	113.6600000000
C9	11.4535970000	48.5880780000	114.8802730000
C10	11.6430400000	47.4500620000	115.8922560000
C11	11.6922530000	46.0156410000	115.3326320000
N12	10.3221450000	45.5166490000	115.1585550000
C13	9.8272470000	44.9674130000	114.0434550000
N14	10.6203440000	44.2353570000	113.2115410000
N15	8.5609560000	45.1457040000	113.7132840000
P16	-2.7370400000	52.6929820000	116.1180040000
O17	-3.3741610000	53.4561740000	115.0371810000
O18	-1.1430680000	52.6791870000	116.1736530000
O19	-3.1174490000	51.0926370000	116.0116510000
O20	-2.5469680000	48.6521770000	115.7294000000
P21	-2.8132160000	49.7405350000	116.8515710000
O22	-1.3500390000	50.1039770000	117.5020070000

O23	-3.8252520000	49.3532450000	117.8491660000
O24	-3.1090410000	53.1509820000	117.6020050000
C25	-1.3824060000	48.6247620000	114.8417800000
C26	-1.0515520000	47.1582180000	114.5523770000
O27	-1.9165770000	46.7154800000	113.5076710000
C28	0.4116520000	46.8827150000	114.1975760000
S29	1.6866880000	47.7504400000	115.0144510000
C30	0.7294590000	45.8809240000	113.3387530000
S31	2.3991200000	45.4978700000	112.9657370000
C32	-0.3163480000	45.0922620000	112.5925040000
N33	-0.2447960000	45.4375400000	111.1793460000
C34	-1.3729270000	45.1599540000	110.4005830000
C35	-1.2400630000	45.1759730000	108.9698460000
O36	-0.2522700000	45.4770120000	108.3085290000
N37	-2.4517940000	44.8225960000	108.3117190000
C38	-3.5934830000	44.4669440000	108.9654410000
N39	-4.6870360000	44.1459810000	108.1700060000
N40	-3.7014630000	44.4547730000	110.2658960000
C41	-2.5899510000	44.8493350000	110.9726600000
N42	-2.7279000000	44.8314220000	112.3508030000
C43	-1.7118780000	45.3286270000	113.2224110000
P44	3.6439640000	47.3369870000	104.8320080000
O45	2.7077530000	48.3967040000	104.4301510000
O46	3.2554200000	46.4253750000	106.0863920000
O47	5.1143750000	47.9418510000	105.2683870000
O48	6.3870100000	48.2999130000	107.4905020000
P49	6.4427800000	47.4882360000	106.1147420000
O50	6.0409650000	45.9839860000	106.4790040000
O51	7.6989740000	47.7072430000	105.3781960000
O52	3.9469640000	46.2479860000	103.7250060000
C53	5.4460030000	48.0626790000	108.5661930000
C54	5.8415390000	48.9926980000	109.7173610000
O55	5.6440260000	50.3016120000	109.2045870000
C56	5.0625420000	48.7436240000	111.0114470000
S57	5.1150050000	47.1616000000	111.7776230000
C58	4.4070370000	49.7505080000	111.6389250000
S59	3.5703910000	49.5101670000	113.1554900000
C60	4.3996850000	51.1619100000	111.0898100000
N61	3.1595290000	51.4408630000	110.3678950000
C62	3.2114410000	52.3546470000	109.3112140000
C63	1.9970100000	52.6477000000	108.6019410000
O64	0.8985430000	52.1178650000	108.7581710000
N65	2.1738440000	53.6388680000	107.5996750000
C66	3.3670930000	54.2463140000	107.3332950000
N67	3.3729610000	55.1939840000	106.3070080000
N68	4.4692010000	53.9447730000	107.9585040000
C69	4.3870550000	52.9565230000	108.9086170000
N70	5.5816730000	52.6097470000	109.5289710000
C71	5.6393350000	51.3405430000	110.1865430000
H72	2.6026430000	55.8539350000	106.3440510000

H73	4.2675070000	55.6707850000	106.2782400000
H74	1.3628600000	53.7972060000	107.0136390000
H75	2.5686760000	50.6376200000	110.1876000000
H76	6.4056390000	52.8706560000	109.0010490000
H77	6.5585530000	51.2985080000	110.7865330000
H78	4.5023150000	51.8733710000	111.9204360000
H79	-4.0497050000	53.3858300000	117.6697200000
H80	-0.7923480000	51.9423690000	116.7127020000
H81	-1.2757500000	49.7151220000	118.3890980000
H82	-1.6410930000	49.1491090000	113.9192720000
H83	-0.5413240000	49.1150340000	115.3328680000
H84	-1.2884070000	46.6009940000	115.4778050000
H85	-3.6798280000	44.8246670000	112.6908550000
H86	0.2051220000	46.3245020000	110.9812810000
H87	-4.4763340000	43.5857680000	107.3517600000
H88	-5.4368080000	43.7483940000	108.7237650000
H89	-2.4278420000	44.9149110000	107.3029230000
H90	-0.1119580000	44.0144940000	112.6933230000
H91	-1.7668740000	44.7776910000	114.1775430000
H92	4.0530460000	45.9608100000	106.4197020000
H93	3.9696400000	46.6495340000	102.8410070000
H94	6.5628890000	45.2835270000	107.0050200000
H95	4.4301190000	48.2968210000	108.2326780000
H96	5.4994110000	47.0151700000	108.8791990000
H97	6.9160820000	48.8349740000	109.9224290000
H98	5.1270300000	52.6274100000	115.8468620000
H99	5.2547740000	51.5304520000	117.2500810000
H100	5.6229000000	49.9220870000	115.6190930000
H101	3.4719380000	44.4680560000	115.5473990000
H102	4.9985920000	44.1766310000	116.3889730000
H103	7.9735300000	45.7908180000	114.2377350000
H104	8.1255880000	44.4948370000	113.0464490000
H105	10.1361570000	43.7614650000	112.4554910000
H106	11.3931630000	43.7327230000	113.6244580000
H107	9.6020440000	46.0067520000	115.7024490000
H108	12.2122470000	45.9793870000	114.3687000000
H109	12.2307100000	45.3618300000	116.0299730000
H110	12.5643900000	47.6237390000	116.4623850000
H111	10.8329150000	47.4793030000	116.6338470000
H112	12.4328710000	48.9315600000	114.5250100000
H113	11.0093540000	49.4387270000	115.4093730000
H114	9.6845950000	47.6361010000	113.7923130000
H115	11.1889140000	47.8760020000	112.8117850000
H116	5.7154790000	42.0495700000	108.4495470000
H117	4.7267860000	43.3928330000	107.8083690000
H118	6.6472590000	44.2427780000	108.8903270000
C119	8.1079990000	43.2150000000	107.9359970000
C120	6.8760000000	51.0000000000	114.3990020000
N121	6.1349170000	50.7961830000	115.5181600000
C122	5.7769600000	51.9129790000	116.3690100000

H123	6.6778680000	52.4501630000	116.6824650000
H124	8.8734990000	43.9649010000	108.1559660000
H125	8.2957390000	42.8161000000	106.9341260000
H126	8.1910890000	42.3958240000	108.6663540000
H127	5.7809990000	42.4766220000	106.7159400000
H128	4.4127790000	42.6968450000	113.9047850000
H129	3.5077870000	42.1373620000	115.2348280000
C130	5.5748210000	41.7254490000	115.1945050000
O131	5.4812520000	40.5002050000	115.2191240000
N132	6.7754890000	42.3708210000	115.2419230000
C133	7.8949990000	41.6539990000	114.7259980000
C134	7.7050970000	41.0676900000	113.2842800000
C135	7.0059340000	42.0117020000	112.3519540000
N136	7.5565860000	43.2173110000	111.9352120000
C137	5.7004450000	41.9472030000	111.9258940000
C138	6.5807240000	43.8771120000	111.3250910000
N139	5.4478690000	43.1346680000	111.2647180000
H140	6.7674350000	43.3686130000	115.0628030000
H141	8.7484870000	42.3416950000	114.7224940000
H142	8.1533640000	40.8222520000	115.3899960000
H143	8.6932000000	40.7796790000	112.9009000000
H144	7.1022410000	40.1607210000	113.3683910000
H145	4.5321070000	43.5070510000	111.0401690000
H146	6.6156040000	44.9109200000	111.0079020000
H147	4.9450200000	41.1896450000	112.0664110000
N148	8.4648640000	49.2539240000	113.9052040000
C149	7.1470000000	49.7700000000	113.5680010000
O150	7.3535880000	52.0956190000	114.1074450000
C151	9.6240670000	49.3716270000	113.1651250000
O152	9.7700370000	50.0996090000	112.1989150000
H153	8.4480510000	48.6070970000	114.6847620000
H154	6.4173000000	48.9741700000	113.7209910000
H155	7.1537520000	50.0553260000	112.5167030000
H156	5.8372960000	46.9960460000	116.2360640000
C157	7.0527690000	46.3037080000	116.4921360000
O158	7.9692500000	46.9077760000	115.9094400000
O159	6.8919680000	45.4194130000	117.2943720000

Coordinates for the structure in **Figure 4.**

Mo1	3.9182373828	47.1247654253	113.9109911052
S2	4.7422757506	47.7596031211	115.8362907857
C3	4.4281362861#	42.6296477860#	114.9024044183#
C4	4.5401685472	44.1116662467	115.3342120436
S5	5.2547942691	45.0659019770	113.9124831944
N6	6.7823427651	43.8579616887	107.9369659334
C7	5.6899656155#	42.8799862102#	107.7290038780#
C8	10.6099993848#	48.2449997390#	113.6600003235#
C9	11.3913783108	48.6112923433	114.9187437179

C10	11.6430397884#	47.4500619743#	115.8922558655#
C11	11.8657766809	46.0800119674	115.2172445891
N12	10.5640734159	45.4896080089	114.9116878053
C13	10.1618245390	44.9216566839	113.7748500264
N14	11.0537083472	44.4811532478	112.8326645323
N15	8.8621670327	44.7524173802	113.5737416938
P16	-2.7370399796#	52.6929821785#	116.1180039686#
O17	-3.3631367491	53.4625458532	115.0350964899
O18	-1.1423599857	52.6628466325	116.1785742852
O19	-3.1388490254	51.0993946419	116.0193736039
O20	-2.4948021210	48.6903940577	115.6918705793
P21	-2.8064258905	49.7401377547	116.8370979796
O22	-1.3500389618#	50.1039769150#	117.5020068725#
O23	-3.8138276200	49.3170339567	117.8237521536
O24	-3.1090410539#	53.1509819276#	117.6020050089#
C25	-1.2836989698	48.6953924642	114.8730780417
C26	-0.9667432145	47.2419805113	114.5341022070
O27	-1.8720030824	46.8469961936	113.5088895103
C28	0.4881273941	46.9679551024	114.1393065259
S29	1.7850016467	47.6331029330	115.0952017766
C30	0.7690057687	46.0241775526	113.1982517111
S31	2.3911847518	45.5288161960	112.8416524105
C32	-0.3109264360	45.3466441182	112.4029382011
N33	-0.3228997008	45.9060633023	111.0581150982
C34	-1.4526713418	45.5620433077	110.2928047661
C35	-1.3840849105	45.6499032308	108.8588635222
O36	-0.4836378278	46.0998661491	108.1635497290
N37	-2.5709543185	45.1529594667	108.2347820484
C38	-3.6237002697	44.6253225822	108.9178379565
N39	-4.6870359062#	44.1459810454#	108.1700060642#
N40	-3.6854830157	44.5798687088	110.2210320570
C41	-2.6043882256	45.0925939236	110.8946352199
N42	-2.6999379783	45.0195316116	112.2707606983
C43	-1.6653252279	45.4804421923	113.1303234249
P44	3.6439641424#	47.3369869881#	104.8320080603#
O45	2.7124102168	48.3979611973	104.4214270154
O46	3.2550630805	46.4153364783	106.0789167950
O47	5.1092397138	47.9458214533	105.2753087944
O48	6.4490259032	48.3130105626	107.4512883949
P49	6.4560365685	47.4787588014	106.0860501860
O50	6.0409647926#	45.9839856184#	106.4790042395#
O51	7.6929107021	47.6698320413	105.3090480203
O52	3.9469640358#	46.2479860534#	103.7250059572#
C53	5.4861227828	48.1461469646	108.5224564161
C54	6.0184047687	48.9085812389	109.7469345334
O55	6.0594983749	50.2860050058	109.3733151103
C56	5.1740217117	48.6174929176	110.9820501985
S57	5.4709190902	47.1535582911	111.8932569479
C58	4.2454712752	49.4948920301	111.4147884551
S59	3.2517668309	49.1775091553	112.8163054828

C60	4.0793860786	50.8180258046	110.7176019109
N61	3.2164662460	50.7081115544	109.5437249346
C62	3.1976948988	51.8699470199	108.7501713103
C63	2.0363221780	52.1738624931	107.9704935885
O64	0.9727860277	51.5540050908	107.9461361558
N65	2.2048260961	53.3355223415	107.1660374460
C66	3.3343781508	54.1012823596	107.1709134886
N67	3.3729610824#	55.1939841274#	106.3070078652#
N68	4.3688811939	53.8344604770	107.9171279108
C69	4.2943961631	52.7099944920	108.6981031605
N70	5.3772979939	52.4927741031	109.5287115618
C71	5.4837604046	51.2581838147	110.2424786216
H72	2.5178279762	55.7388987521	106.2691827294
H73	4.1804606151	55.7732713434	106.5080582366
H74	1.4431835627	53.5190207442	106.5236804112
H75	2.2805115925	50.3653388733	109.7409119912
H76	6.2386597476	52.9464924592	109.2538265224
H77	6.1262532862	51.4176386141	111.1173484397
H78	3.7244434681	51.5767751153	111.4347344600
H79	-4.0452365616	53.4038310314	117.6670221448
H80	-0.8052770533	51.9346910513	116.7384072515
H81	-1.3238944042	49.7893221591	118.4209222593
H82	-1.4825853612	49.2676198199	113.9639029941
H83	-0.4633975930	49.1453768353	115.4337473552
H84	-1.1776454194	46.6574358115	115.4487163361
H85	-3.6208520851	44.8422590759	112.6453399061
H86	-0.0780572509	46.8919890339	111.0319009197
H87	-4.4413629085	43.6643831689	107.3129836795
H88	-5.3361184200	43.6201740745	108.7430342014
H89	-2.5910308619	45.2586454974	107.2269115631
H90	-0.0927913162	44.2721780272	112.3201369679
H91	-1.6621976073	44.8689037374	114.0486560488
H92	4.0611858317	45.9724135143	106.4221428154
H93	3.9855107638	46.6526771504	102.8430490853
H94	6.5674717318	45.2655234813	106.9819231754
H95	4.5219099199	48.5703848014	108.2288797185
H96	5.3766803964	47.0802927839	108.7540914010
H97	7.0502878027	48.5799991420	109.9371836632
H98	5.1306787606	52.6807162521	115.9233307722
H99	5.2908200913	51.5142540390	117.2637816045
H100	5.5288425673	49.9507149401	115.5508601744
H101	3.5452832326	44.5095373070	115.5397660581
H102	5.1650731541	44.2968781860	116.2135830810
H103	8.2431282915	44.9039141532	114.3883769827
H104	8.4833434188	44.1700093613	112.8106339353
H105	10.6636175622	44.3036808284	111.9162824916
H106	11.9620756838	44.9229899297	112.8162880219
H107	9.7739655240	45.9519570063	115.4201269895
H108	12.4713268700	46.2042436862	114.3079828908
H109	12.4223884249	45.4078290112	115.8828180989

H110	12.5225204721	47.6762580206	116.5093682955
H111	10.7961585375	47.3524672147	116.5773713470
H112	12.3491501754	49.0478498936	114.6059226215
H113	10.8583149077	49.4010454677	115.4592829660
H114	9.6845950215#	47.6361009014#	113.7923129442#
H115	11.2194633602	47.8893593954	112.8283044083
H116	5.7040715084	42.0763581948	108.4784435599
H117	4.7290432918	43.4012007673	107.7781381894
H118	6.6481402743	44.2650641933	108.8614398242
C119	8.1079993160#	43.2150001247#	107.9359968525#
C120	6.8760004867#	51.0000003114#	114.3990021968#
N121	6.0336051485	50.8252152664	115.4499784050
C122	5.7769597665#	51.9129784896#	116.3690103926#
H123	6.7202597810	52.3931321103	116.6454045328
H124	8.8753485679	43.9741330758	108.1140237952
H125	8.2877616441	42.7698435215	106.9524615209
H126	8.1924983825	42.4336185085	108.7056987127
H127	5.7920513056	42.4436106886	106.7308074453
H128	4.4127794353#	42.6968453860#	113.9047849675#
H129	3.4934426288	42.1774101924	115.2501005091
C130	5.5581783885	41.6898107043	115.1383839845
O131	5.4308646333	40.4700249013	114.9970864271
N132	6.7508676516	42.3060056665	115.2580787643
C133	7.8949988574#	41.6539996172#	114.7259980973#
C134	7.7447839825	41.0448435934	113.2900737767
C135	6.9375105509	41.8793408121	112.3421160057
N136	7.3832931875	43.0862723851	111.8222067771
C137	5.6272358789	41.6888894088	111.9725931083
C138	6.3474332164	43.6298628272	111.2083801602
N139	5.2697748180	42.8017231316	111.2325712968
H140	6.8272392285	43.2999153069	115.4583354493
H141	8.7023394061	42.3906626778	114.7208684603
H142	8.2243937796	40.8318715334	115.3773411110
H143	8.7578831364	40.8854159869	112.8988628952
H144	7.2619008798	40.0703005115	113.3815919110
H145	4.3249726177	43.0833536703	111.0048898321
H146	6.2944174878	44.6440700418	110.8379240494
H147	4.9334107397	40.9003076608	112.2174982438
N148	8.4591270471	49.2500334328	113.9175567708
C149	7.1469996949#	49.7699996974#	113.5680008492#
O150	7.4298351482	52.0764865917	114.1755365199
C151	9.6038846592	49.3505760265	113.1486346141
O152	9.7146707493	50.0461813644	112.1518350474
H153	8.4553121535	48.5977507090	114.6998528238
H154	6.4070321009	48.9852269330	113.7112502512
H155	7.1712895594	50.0553101036	112.5148064767
C156	7.6850208019	46.1266384916	116.4196158926
O157	8.5427279253	46.9591139678	115.9982989951
O158	7.6057649238	44.9165024042	116.0809854551
H159	6.9508085185	46.4970607257	117.1619691824
